# Prognostic factors for the evolution and reversibility of chronic rejection in pediatric liver transplantation

**DOI:** 10.6061/clinics/2016(04)07

**Published:** 2016-04

**Authors:** Ana Cristina Aoun Tannuri, Fabiana Lima, Evandro Sobroza de Mello, Ryan Yukimatsu Tanigawa, Uenis Tannuri

**Affiliations:** Faculdade de Medicina da Universidade de São Paulo, Instituto da Criança, Unidade Transplante Hepático, São Paulo/SP, Brazil

**Keywords:** Chronic Rejection, Cyclosporine, Pediatric Liver Transplantation, Tacrolimus, Rejection, Mycophenolate Mofetil

## Abstract

**OBJECTIVE:**

Chronic rejection remains a major cause of graft failure with indication for re-transplantation. The incidence of chronic rejection remains high in the pediatric population. Although several risk factors have been implicated in adults, the prognostic factors for the evolution and reversibility of chronic rejection in pediatric liver transplantation are not known. Hence, the current study aimed to determine the factors involved in the progression or reversibility of pediatric chronic rejection by evaluating a series of chronic rejection cases following liver transplantation.

**METHODS:**

Chronic rejection cases were identified by performing liver biopsies on patients based on clinical suspicion. Treatment included maintaining high levels of tacrolimus and the introduction of mofetil mycophenolate. The children were divided into 2 groups: those with favorable outcomes and those with adverse outcomes. Multivariate analysis was performed to identify potential risk factors in these groups.

**RESULTS:**

Among 537 children subjected to liver transplantation, chronic rejection occurred in 29 patients (5.4%). In 10 patients (10/29, 34.5%), remission of chronic rejection was achieved with immunosuppression (favorable outcomes group). In the remaining 19 patients (19/29, 65.5%), rejection could not be controlled (adverse outcomes group) and resulted in re-transplantation (7 patients, 24.1%) or death (12 patients, 41.4%). Statistical analysis showed that the presence of ductopenia was associated with worse outcomes (risk ratio=2.08, p=0.01).

**CONCLUSION:**

The presence of ductopenia is associated with poor prognosis in pediatric patients with chronic graft rejection.

## INTRODUCTION

Despite advances in the field of liver transplantation, chronic rejection (CR) remains a major cause of graft failure and is a common indication for re-transplantation 1. Although the incidence of CR in adults appears to be decreasing and currently ranges from 2% to 5% 1, the incidence of CR continues to range between 8% and 12% in pediatric series 2–6. The best methods for the prevention and treatment of CR remain elusive, and the CR recurrence rate remains as high as 90% after re-transplantation 7.

CR is diagnosed based on histopathological findings. Loss of bile ducts in more than 50% of portal tracts or the presence of foam cell obliterative arteriopathy in the explanted rejected liver are the main indicators of advanced CR. Loss of bile ducts in less than 50% of portal tracts, biliary duct degeneration, perisinusoidal fibrosis, and inflammation are considered preliminary findings for CR after liver transplantation 8.

Previous reports have implicated several risk factors for CR, including autoimmune disease in the recipient 9,10-11, gender mismatch between donor and recipient 12,13, recipient race 14, cytomegalovirus (CMV) infection 15,16-17, recipient age 1, insufficient immunosuppression 1, number and severity of acute rejection (AR) episodes, and re-transplantation for CR 18. However, not all of the suggested risk factors have been confirmed in subsequent studies, and most studies have only included adult patients. There may be qualitative differences in adult and pediatric immune responses, and the risk factors for CR may differ between pediatric and adult transplant recipients 19.

In the pediatric population, factors such as recipient ethnicity, graft type, presence of autoimmune disease, number of AR episodes, and occurrence of post-transplant lymphoproliferative disease (PTLD) have been identified as predictors of CR 19. However, the prognostic factors for the evolution and reversibility of CR in the pediatric liver transplantation population are not known, as few reports have focused on CR in pediatric patients. Thus, the aim of the present study was to evaluate all cases of biopsy-proven CR after liver transplantation in our center to identify factors related to the progression or reversibility of CR.

## METHODS

The study population included children undergoing primary orthotopic liver transplantation at the Instituto da Criança da Universidade de São Paulo between July 1989 and December 2013. Data were collected by retrospective chart review and included age, gender and race of recipient; type of graft; type of preservation solution used; presence of inherent liver disease; duration between liver transplantation and CR diagnosis; occurrence of AR episodes (number and intensity); use of immunosuppressive therapy (drugs and serial levels at the time of CR diagnosis); presence of CMV and Epstein Barr virus (EBV) infection; occurrence of PTLD; and presence of vascular and biliary complications. The study protocol was previously approved by the Ethical Committee of the University of São Paulo Medical School (Surgery Division). Written informed consent was obtained from the parents of all children enrolled in the study.

**Figure 1:**
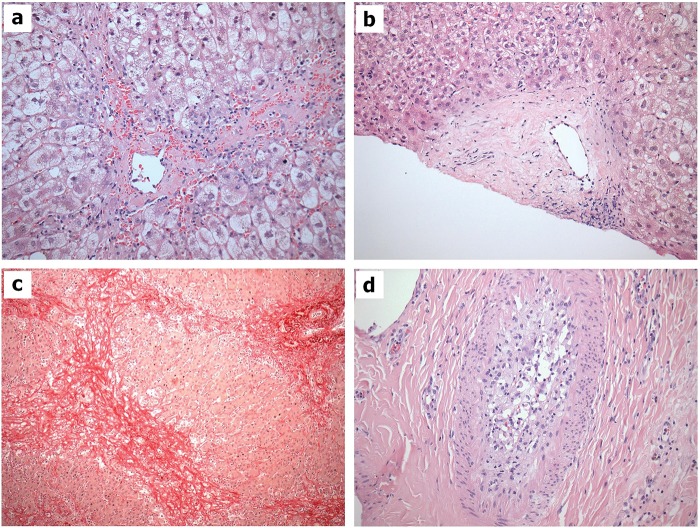
Histopathological findings of liver biopsies. a. Central perivenulitis (hematoxylin-eosin); b. portal space without bile duct (vanishing bile duct) (hematoxylin-eosin); c. lobular fibrosis (picrosirius); d. obliterative arteriopathy (hematoxylin-eosin).

Prior to 2003, primary immunosuppression with cyclosporine and prednisone was used for all pediatric patients. However, from 2003 onwards, cyclosporine was replaced with tacrolimus. The diagnosis of CR was made according to the recommendations of the International Panel of the Banff Schema for Liver Allograft Rejection 8. Liver biopsies were performed on the basis of clinical indication, which was most often elevated liver chemistry. All patients underwent biopsy, and the histopathological findings of all biopsies were evaluated. The main pathological changes indicating CR were vanishing bile duct syndrome and obliterative arteriopathy (Fig. 1). Early stages of CR were indicated by damage to the inter-lobular bile duct, central perivenulitis, perisinusoidal fibrosis, and necrotic inflammation in the central lobule and portal area. Pathological reports containing CR diagnoses were retrieved from our liver pathology database within the study period, and slides were reviewed by three experienced transplantation pathologists (E.S.M, FL and R.Y.T.). Early and late CR diagnoses were confirmed according to previously published criteria 8, and special attention was paid to exclude cases of relapsing autoimmune hepatitis or de novo autoimmune hepatitis 20. Finally, in the last 10 cases, sera was collected from the recipients and verified to contain donor-specific anti-HLA antibodies.

All children with CR diagnosis were maintained on high levels of tacrolimus 10,11-12 and were administered mycophenolate mofetil (MMF) if the tacrolimus level was already high at the time of CR diagnosis. Reversal of histopathological findings and improvement of liver chemistry tests were considered favorable outcomes, whereas indication for re-transplantation or patient death were considered adverse outcomes. The patients were followed for periods ranging from two years to 24 years.

### Statistical Analysis

Data were subjected to descriptive and inferential statistical analysis using Statistical Package for Social Sciences (SPSS) for Windows, version 15.0. For descriptive procedures, we present gross and relative measures (frequencies and percentages) and measures for central tendency (mean) and variability (standard deviation and confidence interval minimum and maximum). The children were divided into two groups according to disease evolution: a favorable outcome (FO) group and an adverse outcome (AO) group. Multivariate analysis was performed to identify potential risk factors for FO or AO.

## RESULTS

Out of the 537 children who underwent liver transplantation at our institution, CR occurred in 29 patients (5.4%).

In 10 patients (10/29, 34.5%), remission of CR with normalization of liver chemistry tests was achieved with immunosuppression (FO group). In 19 patients (19/29, 65.5%), rejection could not be controlled, leading to re-transplantation or death (AO group). The mean data for the FO and AO groups are summarized in Table 1.

Primary immunosuppression was cyclosporine-based in 7 CR cases (24.1%) and tacrolimus-based in 22 (75.9%). The mean tacrolimus serum level at the time of CR diagnosis was 9.7 ng/ml. Ductopenia was present in 11 of the CR cases (37.9%), and foam cell arteriopathy was present in 2 cases (6.9%). Centrilobular changes with fibrosis (68.9%) and inflammation/phlebitis (58.6%) were frequently observed. Most of the CR patients had a previous diagnosis of AR (26 patients, 89.6%), and 17 of them (58.6%) presented two or more episodes of AR. Fifteen CR patients (51.7%) had previous episodes of severe AR. No patients had positive donor-specific anti-HLA antibodies.

In 17 patients (58.6%), the diagnosis of CR was made during the first post-transplant year, and in 7 patients (24.1%), it was within the first 3 months. In terms of final disease evolution, 10 patients (34.5%) survived after being diagnosed with CR, 7 were re-transplanted (24.1%), and 12 (41.4%) patients died.

Multivariate analysis revealed no statistical correlation between CR outcome and recipient age, gender mismatch between donor and recipient, type of graft used, type of preservation solution used, presence of liver disease, time interval between liver transplantation and CR diagnosis, presence of episodes of AR, immunosuppressive regimen at CR diagnosis, presence of CMV and EBV infection, occurrence of PTLD, or presence of any vascular and biliary complications. However, presence of ductopenia was associated with worse outcomes (risk ratio=2.08, *p*=0.01).

Ten (90.9%) of the 11 patients with ductopenic CR were submitted to re-transplantation or died. However, 7 out of the 16 (43.3%) CR patients with no ductopenia presented adverse outcomes.

Eight children underwent re-transplantation. Of these, four did well with no CR relapse (50%). The causes of death in the others were primary non-function (3 patients) and CR relapse (1 patient). In the CR relapse case, the native liver disease was non-syndromic ductopenia. Although high levels of tacrolimus (>10 ng/ml) were maintained post-operatively, CR diagnosis was made less than 1 month after re-transplantation.

## DISCUSSION

Recent advances in surgical techniques and immunosuppression strategies, as well as the implementation of living-related transplant programs and improvements in intensive and postoperative care, have considerably increased pediatric liver transplantation survival rates. CR is an important cause of graft failure, and although the incidence of CR is declining, it remains at 8% to 12% for pediatric liver allograft recipients 2,3,4,5-6. The pathogenesis of CR remains uncertain.

The current study included a heterogeneous group of patients who received cyclosporine or tacrolimus for primary immunosuppression and underwent transplantation over a period spanning 2.5 decades. Our aim was to identify factors related to the progression or reversibility of CR. Although several authors have reported an absence of CR under tacrolimus-based immunosuppression 21,22, the incidence of CR in our population was 5.4%, and most of these CR cases were observed in children already on a tacrolimus regimen. Interestingly, the median tacrolimus levels at the time of CR diagnosis were deemed adequate 9. Furthermore, tacrolimus level was not identified as a prognostic factor in our CR cases; thus, we were not able to replicate the reported significant biochemical improvement and normalized liver function after optimization of baseline immunosuppression. Interestingly, Barbier et al. compared the incidence of CR in patients under high vs. low doses of calcineurin inhibitors and detected no CR in liver graft recipients receiving immunosuppression with low-dose calcineurin inhibitors 23.

Ma et al. investigated the clinical manifestations and pathological features of CR as well the management of CR in 516 adult liver transplant patients 24. Although no typical clinical manifestation was found, their histological findings were concordant with those reported here: vanishing bile duct syndrome and obliterative arteriopathy at late stages, and damage to the inter-lobular bile duct, necrotic inflammation in the central lobule, and inflammatory cell infiltration in the portal area at early stages of CR. They reported an incidence rate of 2.3% for CR, and most of their patients were diagnosed at an early stage. Although they opted for more aggressive management of early CR, including methylprednisolone pulse, OKT3, and anti-thymocyte globulin treatment, we had similar results and reversal indexes when adjusting immunosuppressant doses and mofetil mycophenolate introduction. All of their cases of late CR underwent re-transplantation.

Although AR and CR are considered two different entities with distinct histopathologic patterns, risk factors, and immunological mechanisms, a great majority of our cases of CR (89.6%) were preceded by one or more episodes of AR, with episodes rated as severe in 51.7% of the cases. Prior episodes of AR have already been described as a risk factor for the development of CR in pediatric and adult liver transplantation patients 19. Hence, this relationship may suggest minor dysregulation or perturbation of the immune system or an interaction between the graft and host in affected patients. In this context, centrilobular necroinflammation seems to be a common finding in both conditions. Neil et al. evaluated serial biopsy specimens and failed allografts from 28 patients who underwent re-transplantation for CR to identify histologic features that were present during the early stages of CR. They found that centrilobular necroinflammation associated with portal tract features of AR were present when liver function began to deteriorate. However, the prognosis of CR was not influenced by previous episodes of AR 25. Finally, recent investigations have implicated the importance of de novo donor-specific antibodies in the sera of recipients with CR, especially of HLA-class II type 26.

Despite recent advances in immunosuppression strategies and the introduction of sirolimus therapy in combination with cyclosporine as an effective treatment against CR after liver transplantation 27, there are still limited options for the treatment of this serious complication. In addition, the overall survival without re-transplantation in our study series was only 34.4%. Because the prevalence of CR is relatively low in most centers and its immunological mechanisms are not known, the identification of prognostic factors in patients who develop CR will help predict the evolution of the condition and inform decisions regarding the need for and timing of re-transplantation.

In our study series, the majority of the factors studied (age, gender, recipient race, gender and race of donor, type of graft, type of preservation solution, native liver disease, time between liver transplantation and CR diagnosis, episodes of AR, immunosuppressive therapy, CMV and EBV infection, occurrence of PTLD, vascular and biliary complications) were not correlated with CR prognosis according to multivariate analysis. One possible reason for this finding may be the relatively low number of cases. Collaborations with other centers would increase study numbers and may reveal potential statistical correlations and risk factors for the development of CR in pediatric patients.

In conclusion, CR after pediatric liver transplantation is an important cause of graft failure, with few effective treatment options. The presence of ductopenia is associated with a poor prognosis in CR cases.

## AUTHOR CONTRIBUTIONS

Tannuri AC and Tannuri U performed the surgeries and drafted the manuscript, including the revisions. Lima F, Mello ES, and Tanigawa RY performed the histopathological studies.

## Figures and Tables

**Table 1:** Detailed case data for patients in the favorable and adverse outcomes groups.

	FO group (10 patients) number of patients (%)	AO group (19 patients) number of patients (%)
Age at liver transplantation Median (min-max values)	4 years (1-16 years)	4 years (1-14 years)
Gender	5 F: 5 M	11 F: 8 M
Race	4 Caucasians	9 Caucasians
		4 Mulattos	7 Mulattos
	2 Afro descendants	2 Afro descendants
		1 Indigenous
Underlying disease	4 BA, 2 AHF	11 BA: 2 AIH
		1 AIH, 1 α1ATD	1 CC: 1 NSD
		1 AS, 1 LSD	1 HCVC: 1 AS
		1 PFIC: 1 α1ATD
Donor	7 DD	17 DD
		3 LD	2 LD
Preservation solution	7 Belzer	13 Belzer
		3 HTK	4 HTK
		1 SPS1
		1 Celsior
Ischemia time (mean ± SD)	LD: 1.1±0.4 hrs	LD:1.2±0.3 hrs
	CD: 7.5±3.08 hrs	CD: 7.17±1.64 hrs
Immunosuppressive regimen at CR diagnosis	5 Tac + MMF	10 Tac + MMF
		2 Tac	5 Tac
		2 CyA	3 CyA
		1 CyA + MMF	1 CyA + MMF
CMV infection	0	0
EBV infection	2 (20.0)	3 (15.7)
PTLD	1 (10.0)	2 (10.5)
Vascular complications	3 (30.0)	6 (31.6)
Biliary complications	4 (40.0)	7 (36.8)

(F: female; M: male; BA: biliary atresia; AHF: acute hepatic failure; AIH: autoimmune hepatitis; α1ATD: alpha-1 antitrypsin deficiency; AS: Alagille syndrome; LSD: lysosome storage disease; CC: choledochal cyst; NSD: non-syndromic ductopenia; HCVC: hepatitis C virus cirrhosis; PFIC: progressive familiar intrahepatic cholestasis; PTLD: post-transplant lymphoproliferative disorder; LD: living donor: DD: deceased donor; Tac: tacrolimus; MMF: mycophenolate mofetil; CyA: cyclosporine)
